# Circuit complexity across a topological phase transition

**DOI:** 10.1103/physrevresearch.2.013323

**Published:** 2020

**Authors:** Fangli Liu, Seth Whitsitt, Jonathan B. Curtis, Rex Lundgren, Paraj Titum, Zhi-Cheng Yang, James R. Garrison, Alexey V. Gorshkov

**Affiliations:** 1 Joint Quantum Institute, NIST/University of Maryland, College Park, Maryland 20742, USA; 2 Joint Center for Quantum Information and Computer Science, NIST/University of Maryland, College Park, Maryland 20742, USA; 3 Johns Hopkins University Applied Physics Laboratory, Laurel, Maryland 20723, USA

## Abstract

We use Nielsen’s geometric approach to quantify the circuit complexity in a one-dimensional Kitaev chain across a topological phase transition. We find that the circuit complexities of both the ground states and nonequilibrium steady states of the Kitaev model exhibit nonanalytical behaviors at the critical points, and thus can be used to detect both *equilibrium* and *dynamical* topological phase transitions. Moreover, we show that the locality property of the real-space optimal Hamiltonian connecting two different ground states depends crucially on whether the two states belong to the same or different phases. This provides a concrete example of classifying different gapped phases using Nielsen’s circuit complexity. We further generalize our results to a Kitaev chain with long-range pairing, and we discuss generalizations to higher dimensions. Our result opens up an avenue for using circuit complexity as a tool to understand quantum many-body systems.

## I. INTRODUCTION

In computer science, the notion of computational complexity refers to the minimum number of elementary operations for implementing a given task. This concept readily extends to quantum information science, where quantum circuit complexity denotes the minimum number of gates to implement a desired unitary transformation. The corresponding circuit complexity of a quantum state characterizes how difficult it is to construct a unitary transformation U that evolves a reference state to the desired target state [1,2]. Nielsen and collaborators used a geometric approach to tackle the problem of quantum complexity [3–5]. Suppose that the unitary transformation U(t) is generated by some time-dependent Hamiltonian H(t), with the requirement that $U\left(t_{f}\right)=U$ (where $t_{f}$ denotes the final time). Then, the quantum state complexity is quantified by imposing a cost functional F[H(t)] on the control Hamiltonian H(t). By choosing a cost functional that defines a Riemannian geometry in the space of circuits, the problem of finding the optimal control Hamiltonian synthesizing U then corresponds to finding minimal geodesic paths in a Riemannian geometry [3–5].

Recently, Nielsen’s approach has been adopted in high-energy physics to quantify the complexity of quantum field theory states [6–18]. This is motivated, in part, by previous conjectures that relate the complexity of the boundary field theory to the bulk space-time geometry, i.e., the so-called “complexity equals volume” [19,20] and “complexity equals action” [21,22] proposals. Jefferson *et al.* used Nielsen’s approach to calculate the complexity of a free scalar field [6], and found surprising similarities to the results of holographic complexity. A complementary study by Chapman *et al.*, using the Fubini-Study metric to quantify complexity [23], gave similar results. Several recent works have generalized these studies to other states, including coherent states [8,24], thermofield double states [7,11], and free fermion fields [12–14]. However, the connection between the geometric definition of circuit complexity and quantum phase transitions has so far remained unexplored. This connection is important fundamentally, and it is also intimately related to the long-standing problem of quantum state preparations across critical points [25–27].

In this work, we consider the circuit complexity of a topological quantum system. In particular, we use Nielsen’s approach to study the circuit complexity of the Kitaev chain, a prototypical model exhibiting topological phase transitions and hosting Majorana zero modes [28–33]. Strikingly, we find that the circuit complexity derived using this approach exhibits nonanalytical behaviors at the critical points for both *equilibrium* and *dynamical* topological phase transitions. Moreover, the optimal Hamiltonian connecting the initial and final states must be nonlocal in *real space* when evolving across a critical point. We further generalize our results to a Kitaev chain with long-range pairing, and we discuss universal features of nonanalyticities at the critical points in higher dimensions. Our work establishes a connection between geometrical circuit complexity and quantum phase transitions, and it paves the way toward using complexity as a tool to study quantum many-body systems.

## II. THE MODEL

**FIG. 1.**
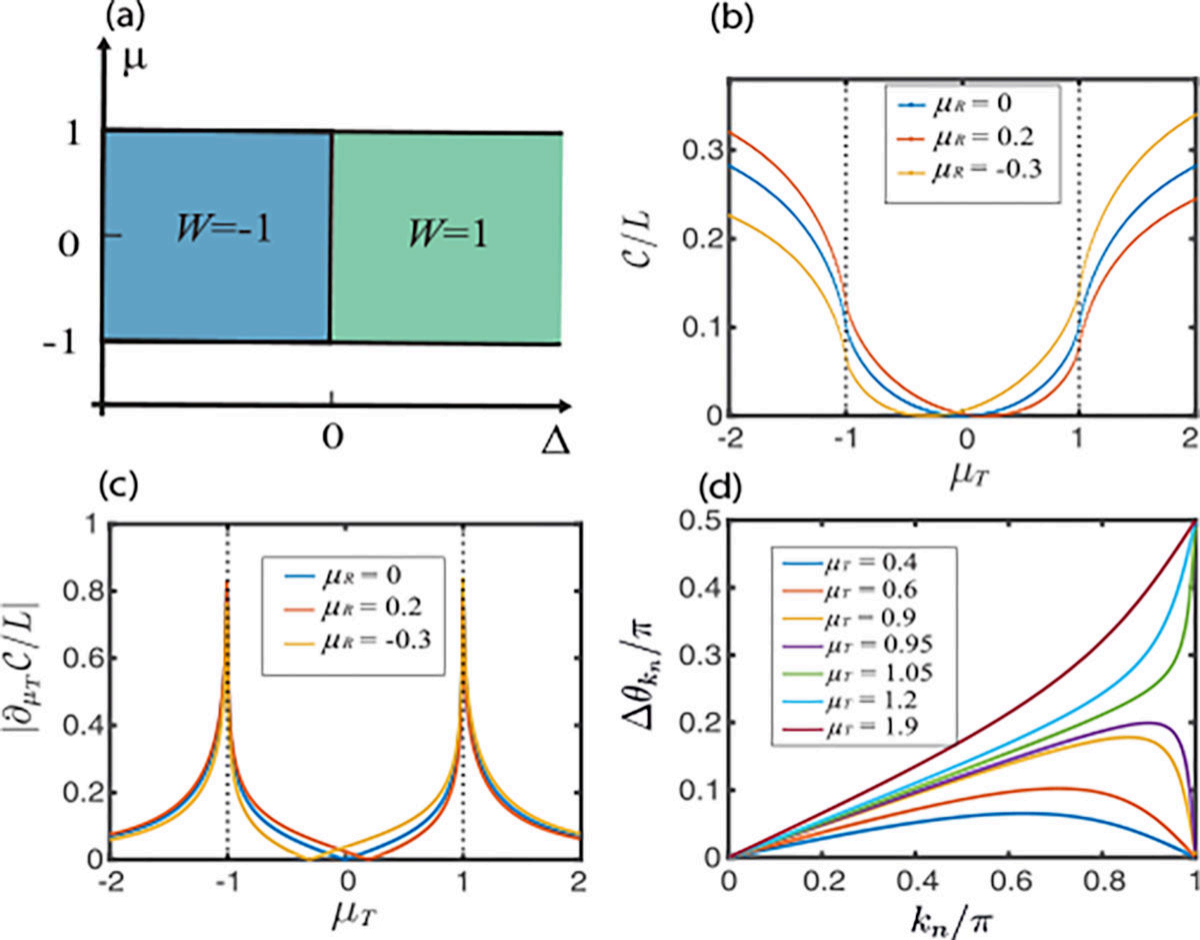
(a) Phase diagram of the Kitaev chain, with W denoting the winding number. (b) Ground-state circuit complexity and (c) its derivative vs target state chemical potential ($\mu_{T}$) for several reference states, each with a different chemical potential $\mu_{R}$. (d) Bogoliubov angle difference, ${\text{Δ}\theta }_{k_{n}}$, for different target ground states, with $\mu_{R}=0$. ${\text{Δ}}_{R}={\text{Δ}}_{T}=1$ for (b)–(d), and L=1000 for (b) and (c).

The one-dimensional (1D) Kitaev model is described by the following Hamiltonian [28,29]:

(1) $$\overset{ˆ}{H}=-\frac{J}{2}\sum_{j=1}^{L}\left({\overset{ˆ}{a}}_{j}^{†}{\overset{ˆ}{a}}_{j+1}+\text{H.c.}\right)-\mu \sum_{j=1}^{L}\left({\overset{ˆ}{a}}_{j}^{†}{\overset{ˆ}{a}}_{j}-\frac{1}{2}\right)\;\;\;+\frac{\text{Δ}}{2}\sum_{j=1}^{L}\left({\overset{ˆ}{a}}_{j}^{†}{\overset{ˆ}{a}}_{j+1}^{†}+\text{H.c.}\right),$$

where J is the hopping amplitude, Δ is the superconducting pairing strength, μ is the chemical potential, L is the total number of sites (assumed to be even), and ${\overset{ˆ}{a}}_{j}^{†}\left({\overset{ˆ}{a}}_{j}\right)$ creates (annihilates) a fermion at site j. We set J=1 and assume antiperiodic boundary conditions (${\overset{ˆ}{a}}_{L+1}=-{\overset{ˆ}{a}}_{1}$). Upon Fourier transforming, Eq. 1 can be written in the momentum basis

(2) $$\overset{ˆ}{H}=-\sum_{k_{n}}\left[\mu +\text{cos}k_{n}\right]\left({\overset{ˆ}{a}}_{k_{n}}^{†}{\overset{ˆ}{a}}_{k_{n}}-{\overset{ˆ}{a}}_{-k_{n}}{\overset{ˆ}{a}}_{-k_{n}}^{†}\right)\;\;\;+i\text{Δ}\;\text{sin}k_{n}\left({\overset{ˆ}{a}}_{k_{n}}^{†}{\overset{ˆ}{a}}_{-k_{n}}^{†}-{\overset{ˆ}{a}}_{-k_{n}}{\overset{ˆ}{a}}_{k_{n}}\right),$$

where $k_{n}=\frac{2\pi }{L}(n+1/2)$ with n=0,1,…,L/2−1. The above Hamiltonian can be diagonalized via a Bogoliubov transformation, which yields the excitation spectrum: $\varepsilon_{k_{n}}=\sqrt{{\left(\mu +\text{cos}k_{n}\right)}^{2}+{\text{Δ}}^{2}{\text{sin}}^{2}k_{n}}$. The ground state of Eq. (1) can be written as

(3) $$\left|{\text{Ψ}}_{\text{gs}}\right\rangle =\prod_{n=0}^{L/2-1}\left(\text{cos}\theta_{k_{n}}-i\text{sin}\theta_{k_{n}}{\overset{ˆ}{a}}_{k_{n}}^{†}{\overset{ˆ}{a}}_{-k_{n}}^{†}\right)|0\rangle ,$$

where $\text{tan}\left(2\theta_{k_{n}}\right)=\text{Δ}\;\text{sin}k_{n}/\left(\mu +\text{cos}k_{n}\right)$. A topological phase transition occurs when the quasiparticle spectrum is gapless [28], as illustrated in Fig. 1(a). The nontrivial topological phase is characterized by a nonzero winding number and the presence of Majorana edge modes [28–33].

## III. COMPLEXITY FOR A PAIR OF FERMIONS

Since Hamiltonian (1) is noninteracting, the ground-state wave function (3) couples only pairs of fermionic modes with momenta $\pm k_{n}$, and different momentum pairs are decoupled. Hence, we first compute the circuit complexity of one such fermionic pair [12–14], and then we obtain the complexity of the full system by summing over all momentum contributions [6,23].

Let us consider the reference (“R”) and target (“T”) states with the same momentum but different Bogoliubov angles: $\left|\psi_{R,T}\right\rangle =\left(\text{cos}\theta_{k}^{R,T}-i\text{sin}\theta_{k}^{R,T}{\overset{ˆ}{a}}_{k}^{†}{\overset{ˆ}{a}}_{-k}^{†}\right)|0\rangle$. Expanding the target state in the basis of $\left|\psi_{R}\right\rangle$ and ${\left|\psi_{R}\right\rangle }_{⊥}$ (i.e., the state orthogonal to $\left|\psi_{R}\right\rangle )$, we have $\left|\psi_{T}\right\rangle =\text{cos}\left(\text{Δ}\theta_{k}\right)\left|\psi_{R}\right\rangle -i\;\text{sin}\left(\text{Δ}\theta_{k}\right){\left|\psi_{R}\right\rangle }_{⊥}$, where $\text{Δ}\theta_{k}=\theta_{k}^{R}-\theta_{k}^{T}$. Now the goal is to find the optimal circuit to achieve the unitary transformation connecting $\left|\psi_{R}\right\rangle$ and $\left|\psi_{T}\right\rangle$:

(4) $$U_{k}=\left[\begin{array}{cc} \text{cos}\left(\text{Δ}\theta_{k}\right) & -ie^{-i\phi }\text{sin}\left(\text{Δ}\theta_{k}\right)\\ -i\text{sin}\left(\text{Δ}\theta_{k}\right) & e^{-i\phi }\text{cos}\left(\text{Δ}\theta_{k}\right) \end{array}\right],$$

where ϕ is an arbitrary phase. Nielsen approached this as a Hamiltonian control problem, i.e., finding a time-dependent Hamiltonian ${𝓗}_{k}(s)$ that synthesizes the trajectory in the space of unitaries [3,4]:

(5) $$U_{k}(s)=\overset{\leftarrow }{𝒫}\text{exp}\left[\int_{0}^{s}dt𝓗(t)\right],{𝓗}_{k}(t)=\sum_{I}Y_{k}^{I}(t)O_{I},$$

with boundary conditions $U_{k}(s=0)=𝟙$ and $U_{k}(s=1)=U_{k}$. Here, $\overset{\leftarrow }{𝒫}$ is the path-ordering operator and $O_{I}$ are the generators of U(2). The idea is then to define a *cost* (i.e., “length”) functional for the various possible paths to achieve $U_{k}$ [3,4,6,12]: $𝒟\left[U_{k}\right]=\int_{0}^{1}ds\sum_{I}{\left|Y_{k}^{I}(s)\right|}^{2}$, and to identify the optimal circuit or path by minimizing this functional. The cost of the *optimal* path is called the circuit complexity 𝒞 of the target state, i.e.,

(6) $$𝒞\left[U_{k}\right]={\text{min}}_{\left\{Y_{k}^{I}(s)\right\}}𝒟\left[U_{k}\right].$$

Following the procedures in Refs. [12–14], one can explicitly calculate the circuit complexity for synthesizing the unitary transformation (4). For quadratic Hamiltonians, it is a simple expression that depends only on the difference between Bogoliubov angles (see Appendix A),

(7) $$𝒞\left(\left|\psi_{R}\right\rangle \to \left|\psi_{T}\right\rangle \right)={\left|\text{Δ}\theta_{k}\right|}^{2}.$$

Note that the complexity 𝒞 for two fermions is at most $\pi^{2}/4$, since $\left|\text{Δ}\theta_{k}\right|\in [0,\pi /2]$. The maximum value is achieved when the target state has vanishing overlap with the reference state.

## IV. COMPLEXITY FOR THE FULL WAVE FUNCTION

Given the circuit complexity for a pair of fermionic modes, one can readily obtain the complexity of the full many-body wave function. The total unitary transformation that connects the two different ground states [Eq. (3)] is

(8) $$\left|{\text{Ψ}}_{\text{gs}}^{T}\right\rangle =\left(\prod_{n=0}^{L/2-1}U_{k_{n}}\right)\left|{\text{Ψ}}_{\text{gs}}^{R}\right\rangle ,$$

where $U_{k_{n}}$, given by Eq. (4), connects two fermionic states with momenta $\pm k_{n}$. By choosing the cost function to be a summation of all momentum contributions [6,12–14], it is straightforward to obtain the total circuit complexity,

(9) $$𝒞\left(\left|{\text{Ψ}}_{\text{gs}}^{R}\right\rangle \to \left|{\text{Ψ}}_{\text{gs}}^{T}\right\rangle \right)=\sum_{k_{n}}{\left|\text{Δ}\theta_{k_{n}}\right|}^{2},$$

where $\text{Δ}\theta_{k_{n}}$ is the difference of the Bogoliubov angles for momentum $k_{n}$. In the infinite-system-size limit, the summation can be replaced by an integral, and one can derive that 𝒞∝L. This “volume law” dependence is reminiscent of the “complexity equals volume” conjecture in holography [19,20], albeit in a different setting.

The circuit complexity given by Eq. (9) has a geometric interpretation, as it is the squared Euclidean distance in a high-dimensional space [34]. The geodesic path (or optimal circuit) in unitary space turns out to be a straight line connecting the two points [i.e., $H_{k}(s)$ independent of s] (Appendix A). In the remainder of this paper, we demonstrate that the circuit complexity between two states is able to reveal both *equilibrium* and *dynamical* topological phase transitions.

We first choose a fixed ground state as the reference state and calculate the circuit complexities for target ground states with various chemical potentials $\mu_{T}$, crossing the phase transition point. The circuit complexity increases as the difference between the parameters of reference and target states is increased [Fig. 1(b)]. More importantly, the complexity grows rapidly around the critical points ($\mu_{T}=\pm 1$), changing from a convex function to a concave function at the critical points. This is further illustrated in Fig. 1(c), where we plot the derivative (susceptibility) of circuit complexity with respect to $\mu_{T}$. The clear divergence at the critical points indicates that circuit complexity is nonanalytical at the critical points (see Appendix B for the derivation), and thus can signal the presence of a quantum phase transition. We emphasize that these features are robust signatures of phase transitions, which do not change if one chooses a different reference state in the same phase [see Figs. 1(b) and 1(c)].

We further plot $\text{Δ}\theta_{k_{n}}$ versus the momentum $k_{n}$ for various target states (with a fixed reference state) in Fig. 1(d). When both states are in the same phase, $\text{Δ}\theta_{k_{n}}$ first increases with momentum and finally decreases to 0 when $k_{n}$ approaches π. In contrast, when $\mu_{T}$ is beyond its critical value, $\text{Δ}\theta_{k_{n}}$ increases monotonically with momentum, and it takes the maximal value of π/2 at $k_{n}=\pi$. This is closely related to the topological phase transition characterized by winding numbers, where the Bogoliubov angles of two different states end up at the same pole (on the Bloch sphere) upon winding half of the Brillouin zone if the states belong to the same phase [29]. Hence, the nonanalytical nature of the circuit complexity is closely related to the change of topological number (and topological phase transition).

Analytically, the derivatives of the circuit complexity (7) can be explicitly recast into a closed contour integral over the complex variable $z=e^{ik}$ (see Appendix B for detailed

derivations). Depending on the parameters of the target states, the poles associated with the integrand are located inside or outside the contour. When the target state goes across a phase transition, the poles sit exactly on the contour, resulting in the divergence of the derivatives of the circuit complexity at critical points (Appendix B). Interestingly, the whole parameter space can be classified into four different phase regimes depending on which poles lie inside the contour (see Fig. 5 in Appendix B), which agrees exactly with the phase diagram shown in Fig. 1(a).

**FIG. 2.**
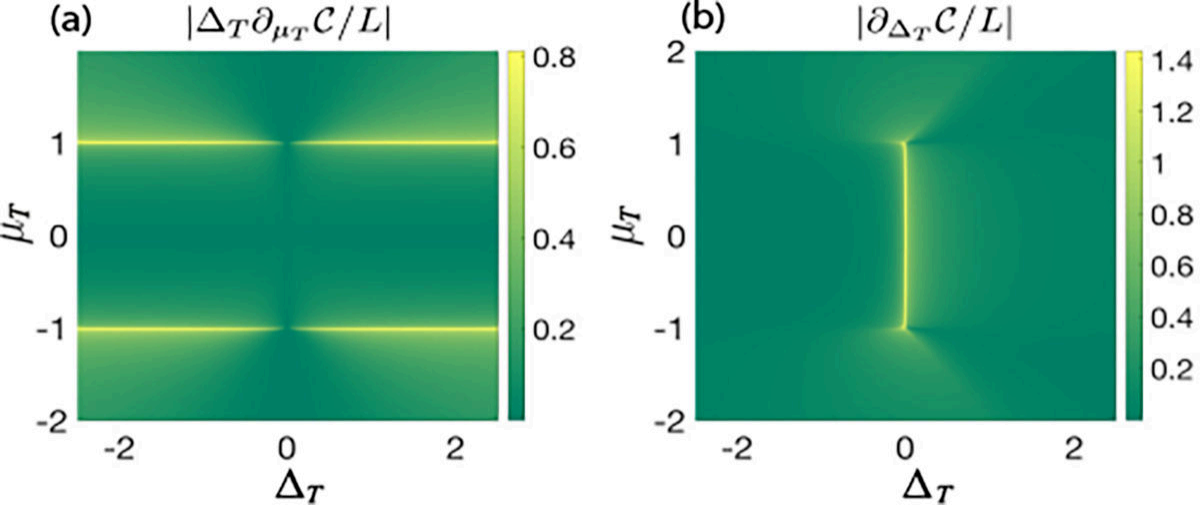
Derivative of circuit complexity as a function of $\mu_{T}$ and ${\text{Δ}}_{T}$. Panel (a) plots the derivative with respect to $\mu_{T}$ (in units of $1/{\text{Δ}}_{T}$), and panel (b) plots the derivative with respect to ${\text{Δ}}_{T}$. The reference state is chosen as the ground state of Eq. (1) with $\mu_{R}=0$ and ${\text{Δ}}_{R}=-1$, and L=1000.

Figures 2(a) and 2(b) show the derivative of circuit complexity with respect to $\mu_{T}$ and ${\text{Δ}}_{T}$ for the whole parameter regime. The derivatives show clear singular behavior at both the horizontal [Fig. 2(a)] and vertical [Fig. 2(b)] phase boundaries. Therefore, by using the first-order derivative of complexity with respect to $\mu_{T}$ and ${\text{Δ}}_{T}$, one can map out the entire equilibrium phase boundaries of the Kitaev chain.

## V. REAL-SPACE LOCALITY OF THE OPTIMAL HAMILTONIAN

Since the ground state [Eq. (3)] is a product of all momentum pairs, the optimal circuit connecting two different ground states corresponds to the following Hamiltonian:

(10) $${\overset{ˆ}{H}}_{c}=\sum_{k>0}{\overset{ˆ}{H}}_{k}=\sum_{k>0}-i\text{Δ}\theta (k){\overset{ˆ}{\psi }}_{k}^{†}\tau_{1}{\overset{ˆ}{\psi }}_{k},$$

where $\tau_{i}$ are the Pauli matrices, and ${\overset{ˆ}{\psi }}_{k}$ denotes the Nambu spinor ψˆk=aˆkaˆ−k†. By taking a Fourier series of the above optimal Hamiltonian, one can show that the Hamiltonian can be written in real space (see Appendix C for details):

(11) $${\overset{ˆ}{H}}_{c}=\sum_{j}\sum_{n=1}^{\infty }\omega_{n}\left({\overset{ˆ}{a}}_{j}{\overset{ˆ}{a}}_{j+n}-\text{H.c.}\right),$$

where $\text{Δ}\theta (k)=2\sum_{n=1}^{\infty }\omega_{n}\text{sin}(nk)$.

One crucial observation is that when the two ground states are in the same phase, Δθ(0)=Δθ(π)=0 [see Fig. 1(d)]; hence the Fourier series of Δθ(k) converges *uniformly*. Therefore, the full series can be approximated by a *finite* order $N^{*}$ with arbitrarily small error. This immediately implies that the real-space optimal Hamiltonian (11) is local, with a *finite range* $N^{*}$. In sharp contrast, if the two states belong to different phases, Δθ(π)=π/2≠Δθ(0)=0; the Fourier series of Δθ(k) converges at most pointwise. Thus the optimal Hamiltonian must be truly *long-range* (nonlocal) in real space (Appendix C), given that the total evolution time is chosen to be a constant [Eq. (5)]. Comparing to previous works on classifying gapped phases of matter using local unitary circuits [35–37], our results provide an alternative approach that has a natural geometric interpretation.

## VI. COMPLEXITY FOR DYNAMICAL TOPOLOGICAL PHASE TRANSITIONS

Dynamical phase transitions have received tremendous interest recently [38–50]. Studies on quench dynamics of circuit complexity have mostly focused on growth rates in the short-time regime [10,15]. Here, we show that the long-time steady-state value of the circuit complexity following a quantum quench can be used to detect *dynamical* topological phase transitions.

We take the initial state to be the ground state of a Hamiltonian ${\overset{ˆ}{H}}_{i},$ and we consider circuit complexity growth under a sudden quench to a different Hamiltonian, ${\overset{ˆ}{H}}_{f}$. The reference and target states are chosen as the initial state $\left|{\text{Ψ}}_{i}\right\rangle$ and time-evolved state |Ψ(t)⟩, respectively. The time-dependent |Ψ(t)⟩ can be written as [51,52]

(12) $$|\text{Ψ}(t)\rangle =\prod_{n=0}^{\frac{L}{2}-1}\left[\text{cos}\left(\text{Δ}\theta_{k_{n}}\right)-ie^{2i\varepsilon_{k_{n}}t}\text{sin}\left(\text{Δ}\theta_{k_{n}}\right){\overset{ˆ}{A}}_{k_{n}}^{†}{\overset{ˆ}{A}}_{-k_{n}}^{†}\right]|0\rangle ,$$

where $\text{Δ}\theta_{k_{n}}$ is the Bogoliubov angle difference between eigenstates of ${\overset{ˆ}{H}}_{i}$ and ${\overset{ˆ}{H}}_{f}$, and $\varepsilon_{k_{n}}$ and ${\overset{ˆ}{A}}_{k_{n}}$ are the energy levels and normal mode operators, respectively, for the postquench Hamiltonian. Similar to the previous derivations, one can obtain the time-dependent circuit complexity,

(13) $$𝒞\left(\left|{\text{Ψ}}_{i}\right\rangle \to |\text{Ψ}(t)\rangle \right)=\sum_{k_{n}}\phi_{k_{n}}^{2}\left(t\right),$$

where $\phi_{k_{n}}(t)=\text{arccos}\sqrt{1-{\text{sin}}^{2}\left(2\text{Δ}\theta_{k_{n}}\right){\text{sin}}^{2}\left(\varepsilon_{k_{n}}t\right)}$.

**FIG. 3.**
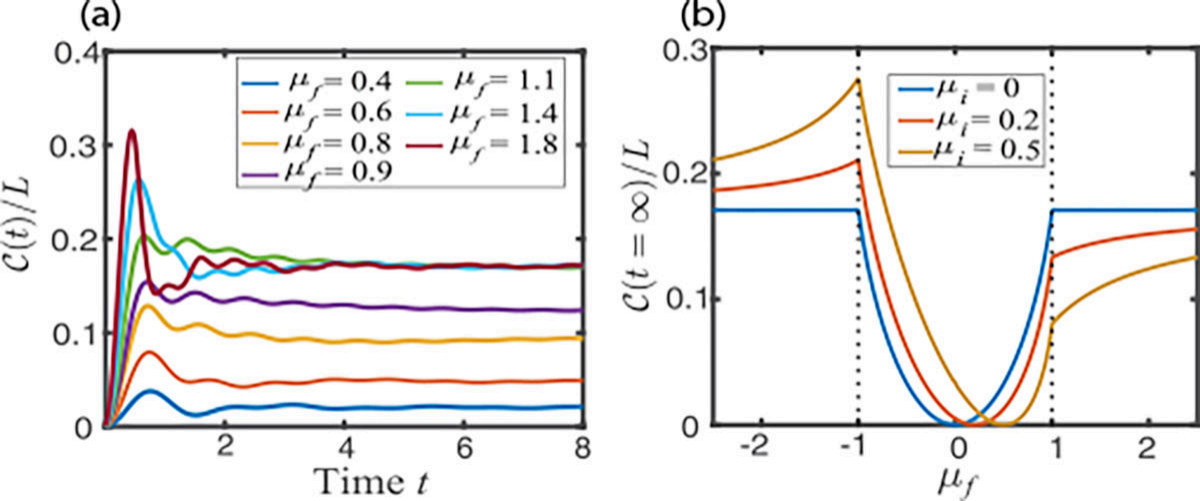
(a) Circuit complexity growth for various postquench chemical potentials, $\mu_{f}$. The initial state (serves as the reference state) is the ground state of Eq. (1) with $\mu_{i}=0$. (b) Steady-state values of complexity vs $\mu_{f}$. The different lines denote different initial/reference states. ${\text{Δ}}_{i}={\text{Δ}}_{f}=1$ and L=1000 in both plots.

As shown in Fig. 3(a), the circuit complexity first increases linearly and then oscillates [9,10,15] before quickly approaching a time-independent value. The steady-state value of circuit complexity increases with $\mu_{f}$ of the postquench Hamiltonian, until the phase transition occurs [Fig. 3(a)]. Figure 3(b) further illustrates the long-time steady-state values of circuit complexity versus $\mu_{f}$ for different initial states. The steady-state complexity clearly exhibits *nonanalytical* behavior at the critical point. This behavior arises because the time-averaged value of $\phi_{k_{n}}(t)$ exhibits an upper bound after the phase transition (see Appendix D), and it is a robust feature of the dynamical phase transition regardless of the initial state.

## VII. GENERALIZATION TO A LONG-RANGE KITAEV CHAIN AND HIGHER DIMENSIONS

**FIG. 4.**
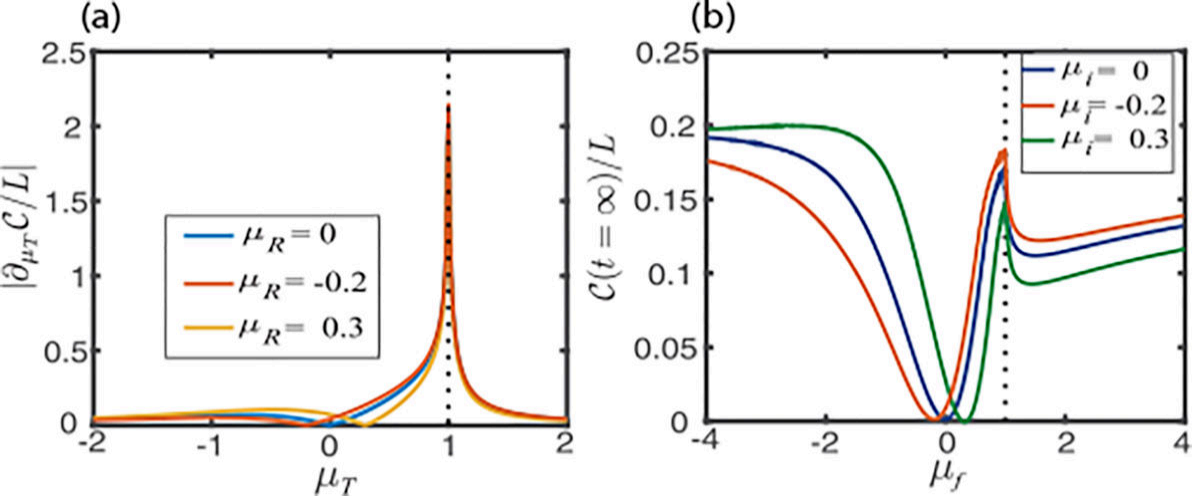
(a) Derivative of circuit complexity with respect to $\mu_{T}$ for three different reference ground states of the long-range Kitaev chain, with ${\text{Δ}}_{R}={\text{Δ}}_{T}=1.3$. (b) Steady-state value of circuit complexity vs $\mu_{f}$ for three different initial ground states, with ${\text{Δ}}_{i}={\text{Δ}}_{f}=1.L=1000$ and α=0 in both plots.

**FIG. 5.**
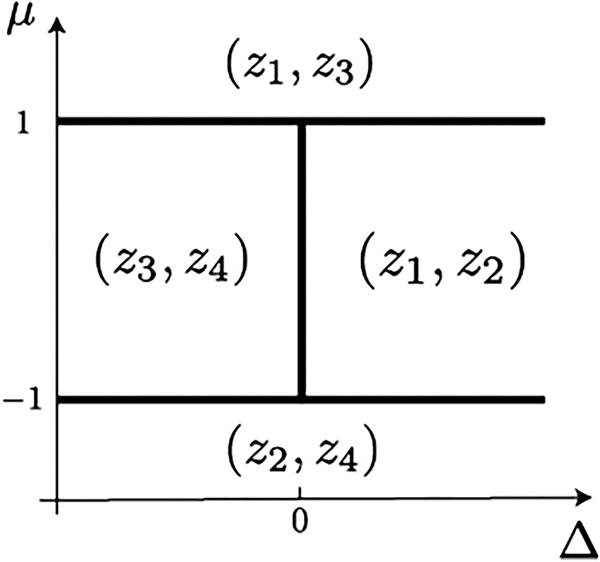
The phase diagram of the Kitaev chain, where in each phase we list which of the two branch points given in Eq. (B5) lies inside the contour integrals in Eq. (B4). The integrals can only diverge at the phase transitions, where the branch points cross the contour.

We further give an example of a Kitaev chain with long-range pairing [53–56]:

(14) $${\overset{ˆ}{H}}_{\text{LR}}=-\frac{J}{2}\sum_{j=1}^{L}\left({\overset{ˆ}{a}}_{j}^{†}{\overset{ˆ}{a}}_{j+1}+\text{H.c.}\right)-\mu \sum_{j=1}^{L}\left({\overset{ˆ}{a}}_{j}^{†}{\overset{ˆ}{a}}_{j}-\frac{1}{2}\right)\;\;\;+\frac{\text{Δ}}{2}\sum_{j=1}^{L}\sum_{\ell =1}^{L-1}\frac{1}{d_{\ell }^{\alpha }}\left({\overset{ˆ}{a}}_{j}^{†}{\overset{ˆ}{a}}_{j+\ell }^{†}+\text{H.c.}\right),$$

where $d_{\ell }=\text{min}(\ell ,L-\ell )$. In contrast to the short-range model, the long-range model with α<1 hosts topological phases with semi-integer winding numbers [53,56]. As one can see, the derivative of ground-state circuit complexity only diverges at $\mu_{T=1}$ [Fig. 4(a)], in contrast with Fig. 1(c). This agrees perfectly with the phase diagram for the long-range interacting model, where a topological phase transition occurs only at μ=1 for α=0 [56]. Figure 4(b) shows the long-time steady-state values of the circuit complexity after a sudden quench. Again, one observes nonanalytical behavior only at $\mu_{T}=1$.

While we have so far restricted ourselves to 1D, the results we found can be readily generalized to higher dimensions [57], for example to p+ip topological superconductors in 2D. The ground-state wave function of a p+ip superconductor essentially takes the same form as Eq. (3), with the momenta now being restricted to the 2D Brillouin zone, and $\text{tan}\left(2\theta_{k}\right)=\left|{\text{Δ}}_{k}\right|/\varepsilon_{k}$, where ${\text{Δ}}_{k}$ and $\varepsilon_{k}$ denote pairing and kinetic terms in 2D. The circuit complexity can still be written as $𝒞=\sum_{k}{\left|\text{Δ}\theta_{k}\right|}^{2}=\frac{L^{2}}{(2\pi {)}^{2}}\int d^{2}k|\text{Δ}\theta (k){|}^{2}$. One can show again that the derivative of the circuit complexity is given by (see Appendix E)

(15) $$\partial_{\mu_{T}}𝒞=\frac{L^{2}}{(2\pi {)}^{2}}\int d^{2}k\frac{\theta^{T}(k)|\text{Δ}(k)|}{E(k{)}^{2}},$$

where $E(k{)}^{2}=\epsilon (k{)}^{2}+|\text{Δ}(k){|}^{2}$, and $\theta^{T}(k)$ denotes the Bogoliubov angle for the target state. It is thus obvious that nonanalyticity happens at the critical point where E(k)=0 (Appendix E).

## VIII. CONCLUSIONS AND OUTLOOK

We use Nielsen’s approach to quantify the circuit complexity of ground states and nonequilibrium steady states of the Kitaev chain with short- and long-range pairing. We find that, in both situations, circuit complexity can be used to detect topological phase transitions. The nonanalytic behaviors can be generalized to higher-dimensional systems, such as p+ip topological superconductors [58,59].

One interesting future direction is to use the geometric approach to quantify circuit complexity when the control Hamiltonians are constrained to be local in real space [37,60,61], and study its connection to quantum phase transitions [25,62–64]. It would also be of interest to investigate the circuit complexity of *interacting* many-body systems. One particular example is the XXZ spin-half chain, whose low-energy physics can be modeled by the Luttinger liquid [65–67]. By restricting to certain classes of gates (i.e., by imposing penalties on the cost function) [3,6], it might be possible to find improved methods to efficiently prepare the ground state of the XXZ model by calculating the geodesic path in gate space.

### Note added:

Recently, we became aware of Ref. [68], which used revivals in the circuit complexity as a qualitative probe of phase transitions in the Su-Schrieffer-Heeger model. In contrast to that work, we have shown that the circuit complexity explicitly exhibits *nonanalyticities* precisely at the critical points for the Kitaev chain. We also became aware of Ref. [57], which numerically studied the complexity of a two-dimensional “d⋅τ” model. By contrast, here we analytically study the “p+ip” model, and we illustrate that the closing of the gap is essential for the nonanalyticity of circuit complexity.

## Figures and Tables

**FIG. 6.**
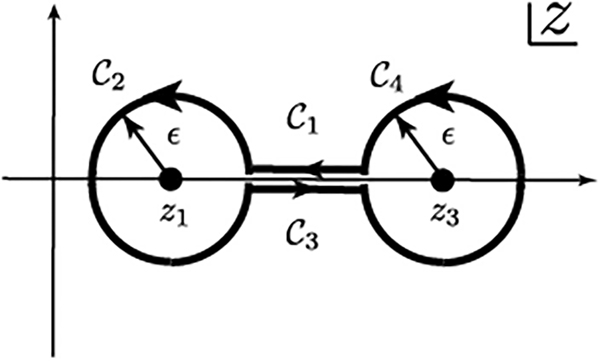
The deformation of the integration contour used to compute the gradients of the circuit complexity in the case $\mu_{T}>1$. There is a branch cut running between the branch points $z_{1}$ and $z_{2}$, where the imaginary part of the integrand is discontinuous and the integrand diverges near the branch points.

**FIG. 7.**
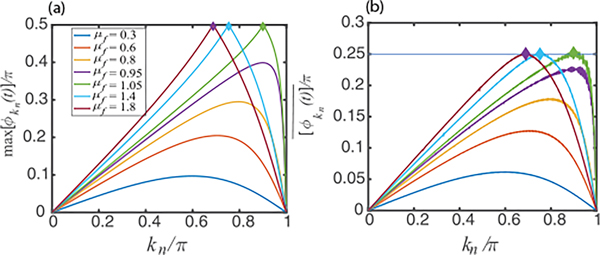
(a) Maximum value of $\phi_{k_{n}}(t)$ vs $k_{n}$ for different postquench Hamiltonian parameters. (b) Time-averaged value of $\phi_{k_{n}}(t)$ vs $k_{n}$ for different postquench Hamiltonian parameters. In both panels, $\mu_{i}=0,{\text{Δ}}_{i}={\text{Δ}}_{f}=1$, and L=1000. The diamond markers denote the expected locations of the maxima across the phase transition, given by solutions to $1+\mu_{f}\text{cos}k_{n}=0$ (see text).

## APPENDIX A: DERIVATION OF CIRCUIT COMPLEXITY FOR A PAIR OF FERMIONS

In this Appendix, we present a detailed derivation of the circuit complexity for a pair of fermions, i.e., Eq. (7). This expression has previously been obtained using different approaches in Refs. [12–14]. To be comprehensive, here we provide a detailed derivation following Ref. [13]. We note that Ref. [12] provides an alternative derivation using a group theory approach.

By taking the derivative with respect to s in Eq. (5), we get the following expression:

(A1)
$$\sum_{I}Y^{I}(s)O_{I}=\left[\partial_{s}U(s)\right]U^{-1}(s),$$

where U(s) is a unitary transformation that depends on s, and we have omitted the label k for notational clarity.

The unitary U(s) can be parametrized in matrix form:

(A2)
$$U(s)=e^{i\beta }\left[\begin{array}{cc} e^{-i\phi_{1}}\text{cos}\omega & e^{-i\phi_{2}}\text{sin}\omega \\ -e^{i\phi_{2}}\text{sin}\omega & e^{i\phi_{1}}\text{cos}\omega \end{array}\right],$$

where $\beta ,\phi_{1},\phi_{2},\omega$ explicitly depend on the parameter s. The above matrix can be expressed in terms of the generators of U(2), which we choose as follows:

(A3)
$$O_{0}=\left[\begin{array}{cc} i & 0\\ 0 & i \end{array}\right],O_{1}=\left[\begin{array}{ll} 0 & i\\ i & 0 \end{array}\right],\;O_{2}=\left[\begin{array}{cc} 0 & 1\\ -1 & 0 \end{array}\right],O_{3}=\left[\begin{array}{cc} i & 0\\ 0 & -i \end{array}\right].$$

Using the relation

(A4)
$$\text{tr}\left(O_{a}O_{b}\right)=-2\delta_{ab},$$

one can extract the strength, $Y^{I}(s)$, of generator $O_{I}$ [cf. Eq. 5] as follows:

(A5)
$$Y^{I}(s)=-\frac{1}{2}\text{tr}\left[\left[\partial_{s}U(s)\right]U^{-1}(s)O_{I}\right].$$

Our cost functional can then be expressed as

(A6)
$$𝒟=\int_{0}^{1}ds\sum_{I}{\left|Y^{I}(s)\right|}^{2}\;\;=\int_{0}^{1}ds\left[{\left(\frac{d\beta }{ds}\right)}^{2}+{\left(\frac{d\omega }{ds}\right)}^{2}+{\text{cos}}^{2}\omega {\left(\frac{d\phi_{1}}{ds}\right)}^{2}\right.\;\;\;\left.+{\text{sin}}^{2}\omega {\left(\frac{d\phi_{2}}{ds}\right)}^{2}\right]..$$

Now, by exploiting the boundary condition at s=0, i.e., U(s=0)=I, we get

(A7)
$$\left(\begin{array}{c} \beta (s=0)\\ \phi_{1}(s=0)\\ \phi_{2}(s=0)\\ \omega (s=0) \end{array}\right)=\left(\begin{array}{c} 0\\ 0\\ \phi_{2}(0)\\ 0 \end{array}\right),$$

where $\phi_{2}(0)$ is an arbitrary phase. Furthermore, we have the boundary condition at s=1,

(A8)
$$U(s=1)=\left[\begin{array}{cc} \text{cos}(\text{Δ}\theta ) & -ie^{-i\phi }\text{sin}(\text{Δ}\theta )\\ -i\text{sin}(\text{Δ}\theta ) & e^{-i\phi }\text{cos}(\text{Δ}\theta ) \end{array}\right],$$

which results in

(A9)
$$\left(\begin{array}{c} \beta (s=1)\\ \phi_{1}(s=1)\\ \phi_{2}(s=1)\\ \omega (s=1) \end{array}\right)=\left(\begin{array}{c} 0\\ 0\\ \pi /2\\ \text{Δ}\theta \end{array}\right).$$

The integrand in Eq. (A6) is a sum of four non-negative terms. Setting $\beta (s)=\phi_{1}(s)=0$ and $\phi_{2}(s)=\pi /2$ minimizes (i.e., sets to zero) three of the four terms without imposing any additional constraints on the minimization of the remaining $(d\omega /ds{)}^{2}$ term. One can then easily check that the linear function w(s)=sΔθ minimizes the remaining term and yields

(A10)
$$𝒞=\int_{0}^{1}ds|\text{Δ}\theta {|}^{2}=|\text{Δ}\theta {|}^{2}.$$

## APPENDIX B: ANALYTICAL DERIVATION OF DIVERGENT DERIVATIVES IN GROUND STATES

In this Appendix, we provide a detailed analytical derivation to show that the first-order derivative indeed diverges at the critical points in the thermodynamic limit. We first derive how the derivative diverges when the reference state is in the trivial phase $\left(\left|\mu_{R}\right|>1\right)$, and then we generalize our results to show how this divergent behavior depends on the particular choice of the reference state. Throughout this Appendix, we assume the reference lies within a given phase, and we allow the target state to approach an arbitrary point in the phase diagram. Our analytical derivations show that these divergences necessarily map out the phase boundary, as illustrated in Figs. 2(a) and 2(b) and Fig. 5 below.

We begin with our general expression for the complexity as a function of our reference and target states. The Bogoliubov angle difference $\text{Δ}\theta_{k}$ for each momentum sector k can be expressed as

(B1)
$$\text{Δ}\theta_{k}=\frac{1}{2}\text{arctan}\frac{\text{sin}k\left[{\text{Δ}}_{R}\mu_{T}-{\text{Δ}}_{T}\mu_{R}+\left({\text{Δ}}_{R}-{\text{Δ}}_{T}\right)\text{cos}k\right]}{\left(\mu_{R}+\text{cos}k\right)\left(\mu_{T}+\text{cos}k\right)+{\text{Δ}}_{R}{\text{Δ}}_{T}{\text{sin}}^{2}k},$$

and the circuit complexity is written in terms of $\text{Δ}\theta_{k}$:

(B2)
$$𝒞/L=\frac{1}{2\pi }\int_{0}^{\pi }{\left|\text{Δ}\theta_{k}\right|}^{2}dk.$$

Note that we have replaced the discrete sum in the main text with an integral for the thermodynamic limit, and written “$𝒞\left(\left|{\text{Ψ}}_{\text{gs}}^{R}\right\rangle \to \left|{\text{Ψ}}_{\text{gs}}^{T}\right\rangle \right)$” as “𝒞” for brevity.

Now we substitute Eq. (B2) into Eq. (B2), and we take the derivatives with respect to $\mu_{T}$ and ${\text{Δ}}_{T}$. We obtain

(B3)
$$\partial_{\mu_{T}}𝒞/L=\frac{{\text{Δ}}_{T}}{4\pi }\int_{-\pi }^{\pi }\frac{\text{Δ}\theta_{k}\text{sin}k}{{\left(\mu_{T}+\text{cos}k\right)}^{2}+{\text{Δ}}_{T}^{2}{\text{sin}}^{2}k}dk,\;\partial_{{\text{Δ}}_{T}}𝒞/L=-\frac{1}{4\pi }\int_{-\pi }^{\pi }\frac{\text{Δ}\theta_{k}\text{sin}k\left(\mu_{T}+\text{cos}k\right)}{{\left(\mu_{T}+\text{cos}k\right)}^{2}+{\text{Δ}}_{T}^{2}{\text{sin}}^{2}k}dk.$$

Here, we have used the fact that these functions are even in k to extend the integrals to −π. In spite of the complicated nature of these integrals, much can be learned about their analytic properties by recasting them as closed contour integrals in the complex plane. Defining the variable $z=e^{ik}$, we find that the integrals take the form

(B4)
$$\partial_{\mu_{T}}𝒞/L=-i{\text{Δ}}_{T}\text{∮}\frac{dz}{2\pi i}\frac{\text{Δ}\theta (z)\left(z^{2}-1\right)}{{\left(z^{2}+2\mu_{T}z+1\right)}^{2}-{\text{Δ}}_{T}^{2}{\left(z^{2}-1\right)}^{2}},\;\partial_{{\text{Δ}}_{T}}𝒞/L=\frac{i}{2}\text{∮}\frac{dz}{2\pi iz}\frac{\text{Δ}\theta (z)\left(z^{2}-1\right)\left(z^{2}+2\mu_{T}z+1\right)}{{\left(z^{2}+2\mu_{T}z+1\right)}^{2}-{\text{Δ}}_{T}^{2}{\left(z^{2}-1\right)}^{2}},$$

where the integration is taken counterclockwise over the contour |z|=1. In this form, we may use the fact that the value of the integrals is entirely determined by the nonanalyticities of the integrand, which are located inside the contour, and that the value of the integration will only diverge if there is a divergence located on the contour.

We proceed by defining the following variables:

(B5)
$$z_{1,a}=\frac{-\mu_{a}+\sqrt{\mu_{a}^{2}+{\text{Δ}}_{a}^{2}-1}}{1+{\text{Δ}}_{a}},\;z_{2,a}=\frac{-\mu_{a}-\sqrt{\mu_{a}^{2}+{\text{Δ}}_{a}^{2}-1}}{1+{\text{Δ}}_{a}},\;z_{3,a}=\frac{-\mu_{a}+\sqrt{\mu_{a}^{2}+{\text{Δ}}_{a}^{2}-1}}{1-{\text{Δ}}_{a}},\;z_{4,a}=\frac{-\mu_{a}-\sqrt{\mu_{a}^{2}+{\text{Δ}}_{a}^{2}-1}}{1-{\text{Δ}}_{a}},$$

where a=R,T. From Eq. (B4), both derivatives contain simple poles at $z_{i,T}$ for i=1,2,3,4, while $\partial_{{\text{Δ}}_{T}}𝒞$ additionally has a simple pole at z=0. Also, using the formula arctan $(z)=\left(\frac{i}{2}\right)\text{ln}\frac{1-iz}{1+iz}$, we can write the Bogoliubov angle as

(B6)
$$\text{Δ}\theta (z)=\frac{i}{4}\text{ln}\left[\frac{\left({\text{Δ}}_{T}+1\right)\left(z-z_{1,T}\right)\left(z-z_{2,T}\right)}{\left({\text{Δ}}_{T}-1\right)\left(z-z_{3,T}\right)\left(z-z_{4,T}\right)}\right.\;\;\;\;\left.\times \frac{\left({\text{Δ}}_{R}-1\right)\left(z-z_{3,R}\right)\left(z-z_{4,R}\right)}{\left({\text{Δ}}_{R}+1\right)\left(z-z_{1,R}\right)\left(z-z_{2,R}\right)}\right].$$

The important fact we will need is that the complex logarithm contains branch cuts running from the zeros to the infinities of its argument; therefore, the $z_{ia}$ are really branch points of the integrand. We now note that the derivatives of the complexity will only diverge if the couplings are tuned to a phase transition. This is because the $z_{i,a}$ can only have unit modulus if we are at one of the phase transitions, and at the phase transitions the branch points cross the contour resulting in a divergent integral; see Fig. 5. In particular, we may characterize the phase diagram in terms of which branch points are inside or outside the contour integral.

In addition, we may actually compute the integrals exactly in certain cases and limits, which allows us to obtain the exact analytic dependence of the divergence on the couplings. As a definite example, we consider the case $\left|\mu_{T}\right|>1$. In this case, there is a branch cut inside the logarithm running from $z_{1,T}$ to $z_{3,T}$, and one outside between $z_{2,T}$ and $z_{4,T}$, and the divergences seen at $\mu_{T}\to 1$ will be due to these branch cuts approaching the contour. In this case, we may entirely factor out the dependence on the reference state from the logarithm and focus on the terms that depend on the target state. We deform the contour so that it skirts the branch cut (see the parametrization into four contours in Fig. 6). A key point here is that the argument of the logarithm is −π upon approaching the branch cut from the bottom-half plane, while it is +π upon approaching it from the top half. Therefore, in the sum of the two contours running along the branch cut, the logarithm simply contributes a phase factor, and we may evaluate the resulting simplified integrand by elementary methods, and for small ϵ we find

(B7)
$$\int_{{𝒞}_{1}}+\int_{{𝒞}_{3}}=\frac{1}{16\sqrt{\mu_{T}^{2}+{\text{Δ}}_{T}^{2}-1}}\text{ln}\left|\frac{\left(z_{3}-z_{2}\right)\left(z_{1}-z_{4}\right)\epsilon^{2}}{\left(z_{1}-z_{2}\right)\left(z_{3}-z_{4}\right){\left(z_{1}-z_{3}\right)}^{2}}\right|.$$

We perform the integral around contour ${𝒞}_{2}$ by writing $z=z_{1}+\epsilon e^{i\theta }$, and integrating from −π<θ<π. At small ϵ, we find

(B8)
$$\int_{{𝒞}_{2}}=-\frac{1}{16\sqrt{\mu_{T}^{2}+{\text{Δ}}_{T}^{2}-1}}\text{ln}\left|\frac{\left({\text{Δ}}_{T}+1\right)\epsilon \left(z_{1}-z_{2}\right)}{\left({\text{Δ}}_{T}-1\right)\left(z_{1}-z_{3}\right)\left(z_{1}-z_{4}\right)}\right|.$$

The computation for contour ${𝒞}_{4}$ is similar, although the phase winds around the other way:

(B9)
$$\int_{{𝒞}_{4}}=-\frac{1}{16\sqrt{\mu_{T}^{2}+{\text{Δ}}_{T}^{2}-1}}\text{ln}\left|\frac{\left({\text{Δ}}_{T}-1\right)\epsilon \left(z_{3}-z_{4}\right)}{\left({\text{Δ}}_{T}+1\right)\left(z_{3}-z_{1}\right)\left(z_{3}-z_{2}\right)}\right|.$$

Finally, taking the sum of all four contours, we find that the ln⁡ϵ divergence in each integral cancels, and we obtain the desired result:

(B10)
$$\partial_{\mu_{T}}𝒞/L=\frac{1}{8\sqrt{\mu_{T}^{2}+{\text{Δ}}_{T}^{2}-1}}\text{ln}\left|\frac{\mu_{T}^{2}-1}{\mu_{T}^{2}+{\text{Δ}}_{T}^{2}-1}\right|\;\;\;+I_{2}\left(\mu_{R},{\text{Δ}}_{R},\mu_{T},{\text{Δ}}_{T}\right),$$

where the function $I_{2}$ depends on $\mu_{R}$ and ${\text{Δ}}_{R}$, but it is analytic as the phase transition is approached. Therefore, when approaching from $\mu_{T}>1$, the quantity $\partial_{\mu_{T}}𝒞/L$ diverges as $\text{ln}\left(\mu_{T}-1\right)/8{\text{Δ}}_{T}$ if ${\text{Δ}}_{T}\neq 0$, but it is analytic if one approaches the multicritical point at ${\text{Δ}}_{T}=0$.

Similar manipulations may be made for $\partial_{{\text{Δ}}_{T}}𝒞/L$ and in other phases. Sometimes the branch cuts take a complicated form in the complex plane so that we cannot reduce the expression into elementary integrals, but we can still deduce the form of the divergence by considering how the contour integrals behave as the branch points cross the contour.

Our final results are summarized as follows. The expression $\partial_{\mu_{T}}𝒞/L$ is always analytic unless $\mu_{T}\to \pm 1$. Near these phase transitions, it diverges as

(B11)
$$\partial_{\mu_{T}}𝒞/L∼\frac{\text{sgn}\left(\mu_{T}\right)}{8\sqrt{\mu_{T}^{2}+{\text{Δ}}_{T}^{2}-1}}\text{ln}\left|\frac{\mu_{T}^{2}-1}{\mu_{T}^{2}+{\text{Δ}}_{T}^{2}-1}\right|,$$

so the divergence is $\text{sgn}\left(\mu_{T}\right)\text{ln}\left|\mu_{T}-1\right|/8{\text{Δ}}_{T}$ if ${\text{Δ}}_{T}\neq 0$, but there is not a divergence at ${\text{Δ}}_{T}=0$.

In contrast, the expression $\partial_{{\text{Δ}}_{T}}𝒞/L$ is analytic whenever ${\text{Δ}}_{T}\neq 0$. In this case, the divergence depends on whether the couplings ($\mu_{T},{\text{Δ}}_{T}$) approach the phase transitions from the topological phase or the trivial phase. If we approach the multicritical points from the trivial phases, we find that $\partial_{{\text{Δ}}_{T}}𝒞/L$ remains analytic. In contrast, if we approach ${\text{Δ}}_{T}=0$ from the topological phases, we find

(B12)
$$\partial_{{\text{Δ}}_{T}}𝒞/L∼\frac{1}{4}\left(1+\frac{\left|\mu_{T}{\text{Δ}}_{T}\right|}{\sqrt{\left|\mu_{T}^{2}+{\text{Δ}}_{T}^{2}-1\right|}}\right)\text{ln}\left|{\text{Δ}}_{T}\right|.$$

In this case, we have a $\text{ln}\;\left|{\text{Δ}}_{T}\right|/4$ divergence when $\left|\mu_{T}\right|<1$, but now we find that the divergence crosses over to $\text{ln}\;\left|{\text{Δ}}_{T}\right|/2$ as we approach the multicritical points.

## APPENDIX C: REAL-SPACE BEHAVIOR OF THE OPTIMAL CIRCUITS

In this Appendix, we show that the real-space optimal circuit behaves differently depending on whether or not the initial and target states are in the same topological phase.

As we have derived in Appendix A, for a single momentum sector k, the circuit complexity is found to be the squared difference between the Bogoliubov angles [Eq. (7)], and the optimal circuit is generated by the following time-independent Hamiltonian:

(C1)
$${𝓗}_{k}=-\text{Δ}\theta_{k}O_{1,k},$$

where $O_{1,k}$ is the same generator given by Eq. (A3) for momentum sector k. Here, we have omitted the time label “s” for simplicity as the circuit is time-independent (and the total evolution time is fixed to be constant 1). As in the main text and following the circuit complexity literature, we have defined ${𝓗}_{k}$ to be anti-Hermitian [Eq. (5)].

Since the ground state of the Hamiltonian is a product of all momentum sectors with k>0, the optimal circuit that generates the evolution between two ground states can be written as

(C2)
$$𝓗=\sum_{k>0}{𝓗}_{k}=\sum_{k>0}-\text{Δ}\theta_{k}O_{1,k}.$$

We are interested in the real-space behavior of the above Hamiltonian. To discern this, we first write the above Hamiltonian in operator form,

(C3)
$$𝓗=\sum_{k>0}{𝓗}_{k}=\sum_{k>0}-i\text{Δ}\theta (k){\overset{ˆ}{\psi }}_{k}^{†}\tau_{1}{\overset{ˆ}{\psi }}_{k},$$

where $\tau_{i}$ are the Pauli matrices, and ${\overset{ˆ}{\psi }}_{k}$ denotes the Nambu spinor,

(C4)
ψˆk=aˆkaˆ−k†.

Utilizing the particle-hole symmetry of the Nambu spinor,

(C5)
$${\overset{ˆ}{\psi }}_{-k}=\tau_{1}{\left({\overset{ˆ}{\psi }}_{k}^{†}\right)}^{T},$$

we can extend the sum in the evolution Hamiltonian to be over the entire Brillouin zone,

(C6)
$$𝓗=\sum_{k}-i\omega (k){\overset{ˆ}{\psi }}_{k}^{†}\tau_{1}{\overset{ˆ}{\psi }}_{k},$$

where ω(k) satisfies

(C7)
ω(k)−ω(−k)=Δθ(k)

for k > 0. In particular, only the odd part of the function contributes since the even part cancels in the $\tau_{1}$ pairing channel.

We now proceed by performing a Fourier series expansion of the function ω(k) over the Brillouin zone. Without loss of generality, we may consider only the odd Fourier series since the even terms will cancel. Thus, we write

(C8)
$$\omega (k)=\sum_{n=1}^{\infty }\omega_{n}\text{sin}(nk)=\frac{\text{Δ}\theta (k)}{2},$$

where the last equality is used to determine the Fourier coefficients.

Our crucial observation is that when the two states are within the same phase, the Fourier sine series for Δθ(k) ought to be uniformly convergent. This can be seen by considering the boundary conditions, which in this case read Δθ(0)=Δθ(π)=0, as shown in Fig. 1(d) in the main text. Thus, if we allow the time-evolved state $\left|{\text{Ψ}}_{T}^{'}\right\rangle$ to be within an arbitrarily small error ϵ to the real target state $\left|{\text{Ψ}}_{T}\right\rangle$, this Fourier series can be accurately truncated to a finite order $N^{*}$ over the entire Brillouin zone.

This is relevant because in real space, the Fourier harmonic $\text{sin}(lk){\overset{ˆ}{\psi }}_{k}^{†}\tau_{1}{\overset{ˆ}{\psi }}_{k}$ is generated by a term involving two fermionic operators separated by l sites. More specifically, as this occurs in the $\tau_{1}$ channel, 𝓗 must involve real-space pairing terms such that

(C9)
$$𝓗=\sum_{j}\sum_{n=1}^{N^{*}}\omega_{n}\left({\overset{ˆ}{a}}_{j}{\overset{ˆ}{a}}_{j+n}-\text{H.c.}\right).$$

The above argument holds when the system size L is taken to be infinite. In such a case, the finite-range interacting evolution Hamiltonian can be regarded as a truly short-range Hamiltonian, and our results imply that the optimal circuit (with constant time or depth) which evolves states within the same phase region is *short-range*.

On the other hand, when the two states are in different phases, the boundary conditions Δθ(π)=π/2≠Δθ(0)=0 obstruct uniform convergence, analogous to the Gibbs phenomenon. In this case, the Fourier sine series may still converge pointwise, but for fixed error the series cannot be truncated to finite order $N^{*}$ over the entire Brillouin zone. In such cases, the optimal evolution Hamiltonian 𝓗 that transforms states between different topological phases must be *long-range* when the evolution time is fixed to be a constant. Again, this is because the longest real-space distance required to generate the evolution Hamiltonian is given by the highest order of Fourier mode appearing in the momentum space series, which now cannot be accurately truncated.

## APPENDIX D: NUMERICAL EVIDENCE FOR NONANALYTICITY OF QUENCH DYNAMICS

In this Appendix, we provide detailed numerical explanations for the nonanalyticity of the long-time steady-state value of the circuit complexity at critical points, as observed in Fig. 3(b).

As derived in the main text, the time-dependent circuit complexity is given by

(D1)
$$𝒞\left(\left|{\text{Ψ}}_{i}\right\rangle \to |\text{Ψ}(t)\rangle \right)=\sum_{k_{n}}\phi_{k_{n}}^{2}(t),$$

where

(D2)
$$\phi_{k_{n}}(t)=\text{arccos}\sqrt{1-{\text{sin}}^{2}\left(2\text{Δ}\theta_{k_{n}}\right){\text{sin}}^{2}\left(\varepsilon_{k_{n}}t\right)}.$$

Then the long-time steady-state complexity is just given by the time-averaged value of the above expression,

(D3)
$$\bar{𝒞\left(\left|{\text{Ψ}}_{i}\right\rangle \to |\text{Ψ}(t)\rangle \right)}=\sum_{k_{n}}\bar{\phi_{k_{n}}^{2}(t)},$$

where the overline denotes time averaging. Because $\phi_{k_{n}}^{2}(t)$ is such a complex expression, it is unknown to us how to derive an analytical function for the time-averaged circuit complexity. Instead, we plot $\bar{\phi_{k_{n}}(t)}$ numerically, and we show that the nonanalyticity indeed occurs at the phase transition.

From the expression of $\phi_{k_{n}}(t)$, it is clear that its value oscillates with time, and it reaches its maximal value (envelope) for each momentum sector $k_{n}$ when $\text{sin}\left(\epsilon_{k_{n}}t\right)=1$. In Fig. 7(a), we plot the maximum value of $\phi_{k_{n}}(t)$ for different postquench Hamiltonian parameters. As the figure clearly shows, when the chemical potential $\mu_{f}$ of the postquench Hamiltonian is below the critical value $\left(\mu_{f}=1\right),\text{max}\left[\phi_{k_{n}}(t)\right]$ is a smooth function of $k_{n}$. However, when $\mu_{f}$ is above the critical value, $\text{max}\left[\phi_{k_{n}}(t)\right]$ exhibits a kink at a certain momentum $k_{n}$, with its maximal value reaching $\frac{\pi }{2}$. To understand this behavior, we can write down the expression for $\text{max}\left[\phi_{k_{n}}(t)\right]$ given the choice of parameters $\mu_{i}=0,{\text{Δ}}_{i}={\text{Δ}}_{f}=1$:

(D4)
$$\text{max}\left[\phi_{k_{n}}(t)\right]=\text{arccos}\left|\frac{1+\mu_{f}\text{cos}k_{n}}{\sqrt{\mu_{f}^{2}+2\mu_{f}\text{cos}k_{n}+1}}\right|.$$

From the above expression, it is clear that when $\mu_{f}<1,\text{max}\left[\phi_{k_{n}}(t)\right]$ is always smaller than π/2; when $\mu_{f}>1,\;\text{max}\left[\phi_{k_{n}}(t)\right]$ can obtain the maximal value of π/2 when $1+\mu_{f}\text{cos}k_{n}=0$. Because one needs to take the absolute value for the arguments of arccos, the quantity $\text{max}\left[\phi_{k_{n}}(t)\right]$ exhibits a kink when reaching π/2, in agreement with Fig. 7(a).

We plot the time-averaged value of $\phi_{k_{n}}(t)$ in Fig. 7(b). Again, we see an upper bound of $\bar{\phi_{k_{n}}(t)}$ when quenching across the critical point. Similar to Fig. 7(a), $\bar{\phi_{k_{n}}(t)}$ reaches its maximal value when $1+\mu_{f}\text{cos}k_{n}=0$, i.e., when $\text{sin}\left(2\text{Δ}\theta_{k_{n}}\right)=1$. For this special momentum sector, the expression for $\phi_{k_{n}}(t)$ can be written as

(D5)
$$\phi_{k_{n}}(t)=\text{arcsin}\left|\text{sin}\left(\varepsilon_{k_{n}}t\right)\right|.$$

Clearly, the time-averaged value of the above expression is just π/4, in agreement with the numerical results shown in Fig. 7(b). Therefore, after the phase transition takes place, the maximal value of $\bar{\phi_{k_{n}}(t)}$ is bounded by π/4. (This feature is independent of the parameters of the prequench Hamiltonian.)

Having revealed this feature of $\bar{\phi_{k_{n}}(t)}$, the nonanalyticity can be understood as follows: as $\mu_{f}$ increases but is still below the phase transition point, the integral of $\bar{\phi_{k_{n}}^{2}(t)}$ increases smoothly with $\mu_{f}$. After reaching the phase transition, $\bar{\phi_{k_{n}}^{2}(t)}$ saturates the bound, and thus the integral’s (circuit complexity’s) dependence on $\mu_{f}$ takes a different form. In particular, for the parameters shown in Fig. 7 [blue line in Fig. 3(b)], the integral (i.e., the circuit complexity) becomes a constant after the phase transition. This leads to a clear nonanalytical (kink) point at $\mu_{f}=1$.

## APPENDIX E: CIRCUIT COMPLEXITY FOR TWO-DIMENSIONAL p+ip TOPOLOGICAL SUPERCONDUCTORS

In this Appendix, we show how our results for the 1D Kitaev chain can be generalized to 2D. In particular, we consider a p+ip topological superconductor for which the Hamiltonian can be written in momentum space as

(E1)
$$\overset{ˆ}{H}=\sum_{k}{\overset{ˆ}{\psi }}_{k}^{†}{𝓗}_{k}{\overset{ˆ}{\psi }}_{k},$$

where the summation is taken over the 2D Brillouin zone, and ψˆk=aˆkaˆ−k+ is the Nambu spinor. The single-particle Hamiltonian takes the following form:

(E2)
$${𝓗}_{k}=\left(\begin{array}{cc} \varepsilon_{k} & {\text{Δ}}_{k}^{*}\\ {\text{Δ}}_{k} & -\varepsilon_{k} \end{array}\right),$$

where $\varepsilon_{k}$ and ${\text{Δ}}_{k}$ denote the kinetic and pairing terms in 2D, respectively. The ground-state wave function can be written as

(E3)
$$\left|{\text{Ψ}}_{\text{gs}}\right\rangle =\prod_{k}\left(\text{cos}\theta_{k}-i\text{sin}\theta_{k}{\overset{ˆ}{a}}_{k}^{†}{\overset{ˆ}{a}}_{-k}^{†}\right)|0\rangle ,$$

where $\text{tan}\left(2\theta_{k}\right)=\left|{\text{Δ}}_{k}\right|/\varepsilon_{k}$. Similar to 1D, the circuit complexity of the full wave function is given by

(E4)
$$𝒞=\sum_{k}{\left|\text{Δ}\theta_{k}\right|}^{2}=\frac{L^{2}}{(2\pi {)}^{2}}\int d^{2}k|\text{Δ}\theta (k){|}^{2},$$

where we have replaced the summation by an integral in the infinite-system limit. In this continuum limit, $\varepsilon (k)\approx \frac{k^{2}}{2m}-\mu$ and $\text{Δ}(k)\approx i\text{Δ}\left(k_{x}+ik_{y}\right)$.

We expect that the nonanalyticity should not depend on the particular choice of initial reference state, so we take $\mu_{R}\to -\infty$ [with $\theta^{R}(k)=0$] for simplicity. This corresponds to the trivial vacuum with no particle. Upon tuning μ, the system undergoes a quantum phase transition into the topological phase at μ=0. Taking the derivative of 𝒞 with respect to $\mu_{T}$, we obtain

(E5)
$$\partial_{\mu_{T}}𝒞=\frac{L^{2}}{(2\pi {)}^{2}}\int d^{2}k2\theta^{T}(k)\partial_{\mu_{T}}\theta^{T}(k)=\frac{L^{2}}{(2\pi {)}^{2}}\int d^{2}k\theta^{T}(k)\partial_{\mu_{T}}\left[\text{arctan}\frac{\left|\text{Δ}(k)\right|}{\varepsilon (k)}\right]=\frac{L^{2}}{(2\pi {)}^{2}}\int d^{2}k\frac{\theta^{T}(k)|\text{Δ}(k)|}{E(k{)}^{2}}.$$

## References

[1] Watrous J, Quantum computational complexity, in Encyclopedia of Complexity and Systems Science, edited by Meyers RA (Springer, New York, 2009), pp. 7174–7201.

[2] Aaronson S, arXiv:1607.05256

[3] Nielsen MA, arXiv:quant-ph/0502070.

[4] Nielsen MA, Dowling MR, Gu M, and Doherty AC, Science 311, 1133 (2006). 16497928 10.1126/science.1121541

[5] Dowling MR and Nielsen MA, arXiv:quant-ph/0701004.

[6] Jefferson RA and Myers RC, J. High Energy Phys 10 (2017) 107.

[7] Yang R-Q, Phys. Rev. D 97, 066004 (2018).

[8] Guo M, Hernandez J, Myers RC, and Ruan S-M, J. High Energy Phys 10 (2018) 011.

[9] Camargo HA, Caputa P, Das D, Heller MP, and Jefferson R, Phys. Rev. Lett 122, 081601 (2019). 10.1103/PhysRevLett.122.081601 30932615

[10] Alves DWF and Camilo G, J. High Energy Phys 06 (2018) 029.

[11] Chapman S, Eisert J, Hackl L, Heller MP, Jefferson R, Marrochio H, and Myers RC, SciPost. Phys 6, 034 (2019).

[12] Hackl L. and Myers RC, J. High Energy Phys 07 (2018) 139.

[13] Khan R, Krishnan C, and Sharma S, Phys. Rev. D 98, 126001 (2018).

[14] Reynolds AP and Ross SF, Class. Quantum Grav 35, 095006 (2018).

[15] Jiang J, Shan J, and Yang J, arXiv:1810.00537

[16] Yang RQ, An YS, Niu C, Zhang CY, and Kim KY, Eur. Phys. J. C 79, 109 (2019).

[17] Yang RQ, An YS, Niu C, Zhang CY, and Kim KY, J. High Energy Phys 03 (2019) 161.

[18] Yang RQ and Kim KY, J. High Energy Phys 03 (2019) 010.

[19] Stanford D. and Susskind L, Phys. Rev. D 90, 126007 (2014).

[20] Susskind L, arXiv:1402.5674

[21] Brown AR, Roberts DA, Susskind L, Swingle B, and Zhao Y, Phys. Rev. Lett 116, 191301 (2016). 10.1103/PhysRevLett.116.191301 27232013

[22] Brown AR, Roberts DA, Susskind L, Swingle B, and Zhao Y, Phys. Rev. D 93, 086006 (2016). 10.1103/PhysRevLett.116.191301 27232013

[23] Chapman S, Heller MP, Marrochio H, and Pastawski F, Phys. Rev. Lett 120, 121602 (2018). 10.1103/PhysRevLett.120.121602 29694101

[24] Caputa P. and Magan JM, Phys. Rev. Lett 122, 231302 (2019). 10.1103/PhysRevLett.122.231302 31298880

[25] Vojta M, Rep. Prog. Phys 66, 2069 (2003).

[26] Caneva T, Fazio R, and Santoro GE, Phys. Rev. B 76, 144427 (2007).

[27] Sørensen AS, Altman E, Gullans M, Porto JV, Lukin MD, and Demler E, Phys. Rev. A 81, 061603(R) (2010).

[28] Kitaev AY, Phys. Usp 44, 131 (2001).

[29] Alicea J, Rep. Prog. Phys 75, 076501 (2012). 10.1088/0034-4885/75/7/076501 22790778

[30] Alicea J, Oreg Y, Refael G, von Oppen F, and Fisher MPA, Nat. Phys 7, 412 (2011).

[31] Sau JD, Lutchyn RM, Tewari S, and Das Sarma S, Phys. Rev. Lett 104, 040502 (2010). 10.1103/PhysRevLett.104.040502 20366693

[32] Oreg Y, Refael G, and von Oppen F, Phys. Rev. Lett 105, 177002 (2010). 10.1103/PhysRevLett.105.177002 21231073

[33] Lutchyn RM, Sau JD, and Das Sarma S, Phys. Rev. Lett 105, 077001 (2010). 10.1103/PhysRevLett.105.077001 20868069

[34] In such a space, each state is represented by one point, with its coordinates labeled by the Bogoliubov angles, i.e., $\left(\theta_{k_{0}},\theta_{k_{1}},\ldots ,\theta_{k_{L/2-1}}\right)$.

[35] Bravyi S, Hastings MB, and Verstraete F, Phys. Rev. Lett 97, 050401 (2006). 10.1103/PhysRevLett.97.050401 17026080

[36] Chen X, Gu Z-C, and Wen X-G, Phys. Rev. B 82, 155138 (2010).

[37] Huang Y. and Chen X, Phys. Rev. B 91, 195143 (2015).

[38] Heyl M. and Budich JC, Phys. Rev. B 96, 180304(R) (2017).

[39] D’Alessio L. and Rigol M, Nat. Commun 6, 8336 (2015). 26424503 10.1038/ncomms9336

[40] Caio MD, Cooper NR, and Bhaseen MJ, Phys. Rev. Lett 115, 236403 (2015). 10.1103/PhysRevLett.115.236403 26684130

[41] Vajna S. and Dóra B, Phys. Rev. B 91, 155127 (2015).

[42] Wilson JH, Song JCW, and Refael G, Phys. Rev. Lett 117, 235302 (2016). 10.1103/PhysRevLett.117.235302 27982622

[43] Wang C, Zhang P, Chen X, Yu J, and Zhai H, Phys. Rev. Lett 118, 185701 (2017). 10.1103/PhysRevLett.118.185701 28524691

[44] Caio MD, Möller G, Cooper NR, and Bhaseen MJ, Nat. Phys 15, 257 (2019).

[45] Heyl M, Pollmann F, and Dóra B, Phys. Rev. Lett 121, 016801 (2018). 10.1103/PhysRevLett.121.016801 30028149

[46] Roy S, Moessner R, and Das A, Phys. Rev. B 95, 041105(R) (2017).

[47] Titum P, Iosue JT, Garrison JR, Gorshkov AV, and Gong Z-X, Phys. Rev. Lett 123, 115701 (2019). 10.1103/PhysRevLett.123.115701 PMC11670880 31573251

[48] Zhang J, Pagano G, Hess PW, Kyprianidis A, Becker P, Kaplan H, Gorshkov AV, Gong ZX, and Monroe C, Nature (London) 551, 601 (2017). 29189781 10.1038/nature24654 PMC6506159

[49] Jurcevic P, Shen H, Hauke P, Maier C, Brydges T, Hempel C, Lanyon BP, Heyl M, Blatt R, and Roos CF, Phys. Rev. Lett 119, 080501 (2017). 10.1103/PhysRevLett.119.080501 28952773

[50] Fläschner N, Vogel D, Tarnowski M, Rem BS, Lühmann D-S, Heyl M, Budich J, Mathey L, Sengstock K, and Weitenberg C, Nat. Phys 14, 265 (2018).

[51] Quan HT, Song Z, Liu XF, Zanardi P, and Sun CP, Phys. Rev. Lett 96, 140604 (2006). 10.1103/PhysRevLett.96.140604 16712060

[52] Kells G, Sen D, Slingerland JK, and Vishveshwara S, Phys. Rev. B 89, 235130 (2014).

[53] Vodola D, Lepori L, Ercolessi E, Gorshkov AV, and Pupillo G, Phys. Rev. Lett 113, 156402 (2014). 10.1103/PhysRevLett.113.156402 25375726

[54] Vodola D, Lepori L, Ercolessi E, and Pupillo G, New J. Phys 18, 015001 (2016).

[55] Patrick K, Neupert T, and Pachos JK, Phys. Rev. Lett 118, 267002 (2017). 10.1103/PhysRevLett.118.267002 28707934

[56] Pezzè L, Gabbrielli M, Lepori L, and Smerzi A, Phys. Rev. Lett 119, 250401 (2017). 10.1103/PhysRevLett.119.250401 29303346

[57] Xiong Z, Yao D-X, and Yan Z, arXiv:1906.11279

[58] Hasan MZ and Kane CL, Rev. Mod. Phys 82, 3045 (2010).

[59] Qi X-L and Zhang S-C, Rev. Mod. Phys 83, 1057 (2011).

[60] Hyatt K, Garrison JR, and Bauer B, Phys. Rev. Lett 119, 140502 (2017). 10.1103/PhysRevLett.119.140502 29053290

[61] Girolami D, Phys. Rev. Lett 122, 010505 (2019). 10.1103/PhysRevLett.122.010505 31012709

[62] Lieb E, Schultz T, and Mattis D, Ann. Phys 16, 407 (1961).

[63] Katsura S, Phys. Rev 127, 1508 (1962).

[64] Perk JHH, Capel HW, Zuilhof MJ, and Siskens TJ, Physica A 81, 319 (1975).

[65] Haldane FDM, Phys JC: Solid State Phys. 14, 2585 (1981).

[66] Voit J, Rep. Prog. Phys 58, 977 (1995).

[67] Rahmani A. and Chamon C, Phys. Rev. Lett 107, 016402 (2011). 10.1103/PhysRevLett.107.016402 21797558

[68] Ali T, Bhattacharyya A, Shajidul Haque S, Kim EH, and Moynihan N, arXiv:1811.05985

